# Working conditions as risk factors for disability retirement: a longitudinal register linkage study

**DOI:** 10.1186/1471-2458-12-309

**Published:** 2012-04-26

**Authors:** Eero Lahelma, Mikko Laaksonen, Tea Lallukka, Pekka Martikainen, Olli Pietiläinen, Peppiina Saastamoinen, Raija Gould, Ossi Rahkonen

**Affiliations:** 1 Hjelt Institute, Department of Public Health, University of Helsinki, Helsinki, Finland; 2 Department of Social Research, Population Research Unit, University of Helsinki, Helsinki, Finland; 3 Finnish Centre for Pensions, Helsinki, Finland

## Abstract

**Background:**

Early retirement due to disability is a public health and work environment problem that shortens working careers. Transition to disability retirement is based on ill-health, but working conditions are also of relevance. We examined the contributions of work arrangements, physical working conditions and psychosocial working conditions to subsequent disability retirement.

**Methods:**

The data were derived from the Helsinki Health Study cohort on employees of the City of Helsinki, Finland. Information on working conditions was obtained from the baseline surveys conducted in 2000, 2001 and 2002. These data were linked with register data on disability retirement and their main diagnoses obtained from the Finnish Centre for Pensions. Follow up by the end of 2008 yielded 525 disability retirement events. The analysed data included 6525 participants and 525 disability retirement events. Hazard ratios (HR) and 95% confidence intervals (95% CI) were calculated from Cox regression analysis.

**Results:**

Several working conditions showed own associations with disability retirement before adjustment. After adjustment for all working conditions, the primary risk factors for all-cause disability retirement were physical workload among women (HR 2.02, 95% CI 1.57-2.59) and men (HR 2.00, 95% CI 1.18-3.38), and low job control among women (HR 1.60, 95% CI 1.29-1.99). In addition, for disability retirement due to musculoskeletal causes, the risk factors were physical workload and low job control. For disability retirement due to mental causes the risk factors were computer work and low job control. Furthermore, occupational class was a risk factor for disability retirement due to all causes and musculoskeletal diseases.

**Conclusions:**

Among various working conditions, those that are physically demanding and those that imply low job control are potential risk factors for disability retirement. Improving the physical working environment and enhancing control over one’s job is likely to help prevent early retirement due to disability.

## Background

Retirement schemes vary from one country to another, and in Finland all employees are covered by mandatory earnings-related pension schemes. Age-based retirement is currently flexible between 63 and 68 years. However, 7.5% of the working age population have retired prematurely due to disability, rendering the true average retirement age 60 years. Thus early retirement due to disability is a serious economic, labour market, work environment and public health problem. Retirement issues have become highly topical in many countries with large post-war baby-boomer generations approaching their mandatory retirement ages. Consequently, due to this demographic transition, the numbers of employees at risk of work disability are rising. To be able to efficiently tackle disability retirement and support longer working careers, a better understanding of the key risk factors contributing to disability retirement is needed.

Our study makes use of a conceptual framework based on analyses of the processes behind retirement and work dis/ability. Beehr’s [1] general model for the retirement process includes both individual resources, health in particular and environmental circumstances, working conditions in particular. Work provides the environment for goal-oriented activity but also contains exposures potentially jeopardising employee health and functioning. Ilmarinen’s [2] multidimensional model for work ability and disability is based equally on individual resources ranging from health to cognitive and other psychological factors as well as environmental circumstances ranging from work to non-work factors. While individual health and functioning are at the core of work ability, the significance of these to work ability is demonstrated in the working environment, which thus sets the standard for the functional resources required [3-5].

If individual health resources are in balance with the requirements of the working environment, work ability can be sustained. However, if health and functioning are undermined, work ability is challenged. Similarly, if physical and mental demands of work increase and working conditions deteriorate, work ability is jeopardised. Prolonged imbalance between health resources and work environment may ultimately lead to loss of work ability and early retirement due to disability. In Finland, a medically confirmed disease affecting work ability is a necessary condition for transition to disability retirement [4,6]. However, in the retirement schemes, work ability is judged in accordance with the conceptual models as a function of both individual health resources and demands of the working environment. A key question - conceptually as well as judicially - is whether an employee is able to continue in his/her current job or whether the process of rehabilitation and disability retirement is triggered [1-4].

The Finnish earnings-related pension schemes cover both age-based retirement and early retirement due to disability. In the public sector, including national and local government, an employee who has an illness, in practice a medically confirmed diagnosis, and who has become incapable of doing his/her own job, is entitled to disability retirement. For a full retirement award, work ability must be reduced by at least three-fifths, and for partial retirement award by two-fifths, over a period of at least one year [4,6]. The emphasis on work ability highlights the need to address the significance of environmental factors in the workplace, work arrangements, physical and chemical exposures, as well as psychosocial working conditions, among the forces driving employees to early retirement due to disability [7,8].

Work arrangements have been examined in a number of studies. Evening and night work among Danish nurses was associated with disability retirement even when sociodemographics, psychosocial and physical working conditions were considered [9]. Transfer from public sector to executive agencies in Britain led to increased work disability among employees when sociodemographics, socioeconomic position and health, but not working conditions, were considered [10]. Among Finnish employees, low employee control over working times predicted disability retirement, in particular due to musculoskeletal diseases [11]. Socioeconomic position, shift work and psychosocial factors, as well as health behaviours and health status, were considered, but physical working conditions were not included. In another Finnish study men working long hours were at high risk of disability retirement whereas men doing shift work were at the lowest risk after considering sociodemographics, socioeconomic position, health status and health behaviours, but not specific working conditions [12].

Psychosocial factors have been examined in several studies. Among Danish women and men, both low decision authority and variation in work particularly among men were associated with disability retirement when physical working conditions and health behaviours were considered [8]. In another Danish study, job dissatisfaction was associated with disability retirement, but other working conditions were not included [13]. After considering sociodemographics and socioeconomic position, a Norwegian study showed a weak association with low job autonomy among men only [14]. In another Norwegian study, job control was associated with disability retirement after considering further psychosocial working conditions, sociodemographics, socioeconomic position and health status [15]. Among Finnish men, mental strain, job dissatisfaction and social support were associated with disability retirement when sociodemographics and socioeconomic position as well as health status and health behaviours, but not other working conditions, were considered [12]. In another Finnish study, burnout was associated with disability retirement even when sociodemographics and socioeconomic position, health status and medication were considered [16]. A further Finnish study showed that job strain continued to be associated with disability retirement when socioeconomic position, health behaviours and health status, but not further working conditions, were considered [17].

Some studies have also examined physical working conditions. Among Finnish men, heavy physical work, uncomfortable working positions and noise were associated with disability retirement after considering sociodemographics and socioeconomic position as well as health status and health behaviours, but not further working conditions [12]. In another study, lifting, static load and uncomfortable working positions were associated with disability retirement after considering socioeconomic position, health behaviours and oxygen intake [18]. These associations held true for all-cause disability retirement and musculoskeletal causes, but not for cardiovascular or mental causes. In a Danish study, physical demands and repetitive work were associated with disability retirement among both women and men after considering health behaviours and psychosocial factors at work [19]. Among Danish nurses, physical demands were associated with disability retirement even when sociodemographics, work arrangements, psychosocial factors and health behaviours were considered [9]. Among Norwegian employees, physical demands were also associated with disability retirement when psychosocial working conditions, sociodemographics, socioeconomic position and health status were considered [15]. In another study among Norwegian employees, physical strain was associated with disability retirement when sociodemographics and socioeconomic position were considered, but further working conditions were not included [14].

Summarising the previous studies confirms that comprehensive work environment frameworks are conspicuously lacking. The studies tend to focus on limited or specific working conditions in addition to considering conventional sociodemographics, socioeconomic position and prior health status as covariates. Thus the relative importance of various subdomains of working conditions and specific conditions cannot be compared. Adjustments for further subdomains of working conditions are at best insufficient, but often fully lacking. Many studies have relied on self-reports, but some have benefitted from reliable register-based data on retirement and risk factors.

Most studies have examined all-cause disability retirement only. Musculoskeletal and mental problems are the main causes of disability retirement, and in Finland, for example, they account for two-thirds of all new events [6]. As suggested by conceptual models [1-3] and previous research, a broad range of different working conditions may contribute to disability retirement and this may vary between the retirement diagnoses. Physical workload and other physical and chemical exposures are likely to influence physical health directly. We expect these exposures to contribute, in particular, to disability retirement due to somatic problems, musculoskeletal diseases in particular. Psychosocial and mental exposures in turn operate mainly through stress mechanism and health behaviours, and are likely to influence mental health. We expect these exposures to contribute predominantly to disability retirement due to mental disorders, depression in particular. Nevertheless, the evidence so far is not unequivocal, and both physical and psychosocial exposures may contribute to disability retirement due to musculoskeletal diseases as well as mental disorders [11,14,16].

The present study attempts to fill existing gaps in our understanding by introducing a framework of work-related risk factors of disability retirement which is broader than those used before. Our general aim was to examine associations of work arrangements and physical and psychosocial working conditions with subsequent disability retirement due to all causes, as well as musculoskeletal and mental causes. The specific aims were:

1) To examine associations of each working condition with disability retirement;

2) To examine associations of each working condition with disability retirement after groupwise adjustment for the specific working conditions within the groups of work arrangements, physical working conditions and psychosocial working conditions;

3) To examine associations of each working condition with disability retirement after adjusting for all studied working conditions simultaneously; and

4) To control the importance of socioeconomic position to the studied associations.

## Methods

### Data sources

The data for this study were derived from the Helsinki Health Study cohort on the staff of the City of Helsinki. The City of Helsinki is the largest employer in Finland, with almost 40 000 employees. It provides many basic services, including social and health care, educational and cultural services, environmental and technical maintenance as well as public administration and transportation. The staff includes hundreds of blue-collar and white-collar occupations [20].

Data on working conditions were obtained from the baseline surveys collected in 2000, 2001 and 2002 from employees who reached the ages of 40, 45, 50, 55 and 60 in each of these years. The total sample included 13 344 employees to whom mail questionnaires were sent. Altogether 8960 employees responded at baseline (response rate 67%). Of the total number of respondents, 80% were women. This corresponds to the gender distribution in the target population of the staff of the City of Helsinki. An analysis of non-response showed that the baseline respondents generally represented the target population, with men, younger employees, manual workers and those with prolonged sick absence, being somewhat underrepresented [20,21].

Data on disability retirement were obtained from the national registers of the Finnish Centre for Pensions, providing complete information of all retirement events and their main diagnoses (ICD-10). Disability retirement events were followed up at the end of 2008 and linked to the baseline survey data using unique personal identification numbers. Participants who, during the follow-up, retired based on age (n = 1170), reached 63 years (n = 204) or died (n = 49), were censored. In the Finnish retirement scheme, pensions granted after the age of 63 years are no longer based on disability but on age alone. The register data were linked to the baseline survey data only for participants who had provided written consent for such linkage (74%). After excluding those not consenting to data linkage (n = 2354), the number of participants included in this study was 6525. Non-response analyses also examined the effect of consenting to data linkage and showed that bias by sociodemographics and sickness absence was unlikely [21].

A medically confirmed diagnosis is required for disability pension in the Finnish pension scheme, and the main retirement diagnoses are contained in the register data. Disability retirement events due to all causes amounted to 525. Of these, 217 were due to musculoskeletal diseases (ICD-10 M00-M99), 143 due to mental disorders (ICD-10 F00-F99) and 165 due to various other causes. The proportion of both musculoskeletal and mental causes of all disability retirement events was 70% among women and 63% among men.

### Working conditions

Following procedures used in our previous work [7], three broad subdomains of working conditions were examined, namely work arrangements, physical working conditions and psychosocial working conditions.

The work arrangements were: (a) Working time categorised by overtime, i.e. those doing more than 40 hours work per week and those doing less; (b) Shift work categorised by those doing shift work and those doing regular day-time work; and (c) Work contract categorised by those with permanent and temporary contracts. Information on work contracts was obtained from the employer’s registers.

Physical working conditions were measured using an inventory developed at the Finnish Institute of Occupational Health [22]. Three factors were derived from 18 dichotomous items using a polychoric correlation matrix. First physical workload included uncomfortable postures, repetitive trunk rotation, repetitive movements, heavy physical exertion or lifting and carrying, and standing and walking, which all loaded on the first factor (Cronbach α 0.82). Second hazardous exposures included dirt and dust, dampness and wetness, noise, solvents or other irritating substances, as well as problems with lighting or temperature, which loaded on the second factor (Cronbach α 0.79). Third computer work included working with a computer display terminal, using a computer mouse and doing sedentary work, which loaded on the third factor (Cronbach α 0.80). The factors were dichotomised closest to the upper quartile to indicate the most adverse working conditions and help compare the relative importance of the specific working conditions with each other [7].

The psychosocial working conditions were: (a) Work stress based on Karasek’s [23] job strain questionnaire and containing two separate dimensions, i.e. nine items on job demands and nine items on job control, and (b) Social support at work using four questions from Sarason’s [24] brief inventory of opportunities to receive help from colleagues or a supervisor. These variables were also dichotomised closest to the upper quartile, indicating the most adverse conditions.

Occupational class was derived from the employer’s registers and completed from questionnaires of those not consenting to data linkage. Occupational class was categorised into managers and professionals, semi-professionals, routine non-manual employees and manual workers. Major occupations in our study include nurses, nurses’ aids, nursery teachers and childminders, home aids, kitchen workers, social workers, office secretaries, teachers and physicians among women, and bus drivers, foremen, teachers, office managers and physicians among men.

### Statistical methods

Prevalence data on adverse working conditions and the distribution of occupational class for women and men included in, and excluded from, the analyses due non-consent for register data linkage were calculated (Table 1). Most of the differences in adverse working conditions between the included and the excluded were modest, and the direction of the difference varied by the working condition. Thus major selection is unlikely among those included in the present analyses.

**Table 1:** Prevalence of adverse working conditions and distribution of occupational class among women and men consenting to data linkage (=Included, n = 6525) and not consenting (=Excluded, n = 2354)

	**Women Included n**			**Men Included n**		
		**% among those included**	**% among those excluded**		**% among those included**	**% among those excluded**
1) Work arrangements:						
Shift work	5083	16	17	1394	22	24
Temporary work contract	5049	12	10	1386	8	9
Working overtime	5049	13	12	1384	22	21
2) Physical working conditions:						
Hazardous exposures	4170	22	25	1230	32	37
Physical workload	4170	28	31	1230	11	17
Computer work	4170	25	25	1230	25	18
3) Psychosocial working conditions:						
Low job control	4967	21	25	1373	24	24
High job demands	4805	23	23	1357	20	19
Low social support	5122	34	26	1402	32	24
4) Occupational class:						
Managers and professionals	1462	29^*^	23^*^	625	45^*^	37^*^
Semi-professionals	986	19	17	281	20	16
Routine non-manual workers	2105	42	45	132	9	13
Manual workers	561	11	15	364	26	33

*Column percentages.

Multiple imputations for missing values on the working conditions were conducted using R software [25]. The imputation process was used to create 10 datasets with missing values replaced by imputed candidates. The analyses were then run on each imputed dataset and the results combined by taking averages of the coefficients and adjusting standard errors for the imputation. The data were assumed missing at random.

Associations of working conditions with disability retirement were examined using Cox proportional hazards models. Models were fitted to yield hazard ratios (HR) and their 95% confidence intervals (95% CI), indicating the risk of disability retirement among those suffering from adverse working conditions. Age at baseline and age at first event (disability retirement, age-based retirement, end of follow up, age 63, death) was used as a dependent variable rendering age adjustment redundant. First, in the unadjusted base model, own effects of each specific working condition were examined. Second, models were fitted, which adjusted for the specific working conditions belonging to each of the three groups of working conditions, namely, work arrangements, physical working conditions and psychosocial working conditions (‘groupwise adjustment’). Third, all working conditions were simultaneously adjusted for each other. Additionally, the own effect of occupational class was shown, and a fully adjusted model including all working conditions together with occupational class, was also fitted. Analyses of all-cause retirement were stratified by gender. In the cause-specific analyses, women and men were pooled due to small numbers of retirement events and gender was included as a covariate. Gender differences were tested by fitting interactions. All analyses were made using R statistical software version 2.11.0.

### Ethical considerations

The Helsinki Health Study protocol has been approved by ethics committees of the Department of Public Health, University of Helsinki, and the City of Helsinki health authorities, Finland.

## Results

### All-cause disability retirement

First, among women, unadjusted base models were fitted to examine own associations of each specific working condition with all-cause disability retirement. There were associations suggesting an increased risk of disability retirement among those doing shift work, suffering from hazardous exposures and heavy physical workload, as well as low job control (Table 2). The hazard ratio was highest for physical workload (HR 2.26, 95% CI 1.82-2.81). Additionally, social support showed a reverse association (HR 0.78, 95% CI 0.63-0.97). Second, groupwise models were adjusted for the specific working conditions within each of the three broad subdomains of working conditions. The associations remained except for hazardous exposures and social support. Third, in the models, mutually adjusting all working conditions for each other physical workload and job control remained associated with disability retirement.

**Table 2:** Associations of working conditions with all-cause disability retirement, unadjusted, adjusted for groups of working conditions (=Groupwise), all working conditions and all variables in analysis

**Women**	**Unadjusted**	**Groupwise adjusted**	**All working conditions adjusted**	**Fully adjusted**
	**HR (95% CI)**	**HR (95% CI)**	**HR (95% CI)**	**HR (95% CI)**
1) Work arrangements:				
Shift work	1.40 (1.10, 1.78)	1.37 (1.07, 1.75)	1.04 (0.81, 1.35)	1.02 (0.79, 1.31)
Temporary work contract	0.92 (0.68, 1.26)	0.90 (0.66, 1.23)	0.96 (0.70, 1.31)	1.03 (0.75, 1.42)
Working overtime	0.75 (0.56, 1.01)	0.78 (0.58, 1.05)	0.91 (0.67, 1.23)	1.02 (0.75, 1.39)
2) Physical working conditions:				
Hazardous exposures	1.60 (1.27, 2.02)	1.13 (0.88, 1.45)	1.10 (0.86, 1.42)	1.06 (0.82, 1.36)
Physical workload	2.26 (1.82, 2.81)	2.22 (1.74, 2.82)	2.02 (1.57, 2.59)	1.73 (1.35, 2.22)
Computer work	0.95 (0.75, 1.22)	1.15 (0.90, 1.49)	1.11 (0.85, 1.45)	1.28 (0.97, 1.69)
3) Psychosocial working conditions:				
Low job control	1.88 (1.53, 2.31)	1.85 (1.50, 2.28)	1.60 (1.29, 1.99)	1.34 (1.07, 1.68)
High job demands	1.05 (0.83, 1.32)	1.10 (0.87, 1.39)	1.06 (0.83, 1.35)	1.13 (0.88, 1.44)
Social support at work	0.78 (0.63, 0.97)	0.85 (0.68, 1.06)	0.89 (0.71, 1.11)	0.92 (0.74, 1.15)
4) Occupational class:				
Managers and professionals	1.00			1.00
Semi-professionals	2.13 (1.49, 3.05)			1.91 (1.32, 2.77)
Routine non-manual workers	2.47 (1.83, 3.32)			1.95 (1.40, 2.71)
Manual workers	4.36 (3.13, 6.08)			3.18 (2.16, 4.68)
**Men**	**Unadjusted**	**Groupwise adjusted**	**All working conditions adjusted**	**Fully adjusted**
	**HR (95% CI)**	**HR (95% CI)**	**HR (95% CI)**	**HR (95% CI)**
1) Work arrangements:				
Shift work	2.20 (1.45, 3.34)	2.17 (1.43, 3.30)	1.38 (0.84, 2.26)	1.16 (0.70, 1.93)
Temporary work contract	0.68 (0.30, 1.55)	0.77 (0.33, 1.75)	0.80 (0.35, 1.84)	0.84 (0.36, 1.96)
Working overtime	0.81 (0.5, 1.33)	0.81 (0.50, 1.33)	0.88 (0.53, 1.48)	0.95 (0.56, 1.60)
2) Physical working conditions:				
Hazardous exposures	2.47 (1.61, 3.77)	2.14 (1.31, 3.48)	1.69 (0.99, 2.89)	1.63 (0.93, 2.86)
Physical workload	2.80 (1.72, 4.54)	1.91 (1.13, 3.22)	2.00 (1.18, 3.38)	1.75 (1.02, 3.00)
Computer work	1.07 (0.67, 1.72)	1.31 (0.79, 2.17)	1.48 (0.89, 2.48)	1.74 (1.02, 2.97)
3) Psychosocial working conditions:				
Low job control	2.21 (1.49, 3.28)	2.08 (1.39, 3.12)	1.54 (0.99, 2.40)	1.41 (0.90, 2.21)
High job demands	0.62 (0.35, 1.12)	0.68 (0.38, 1.24)	0.67 (0.36, 1.23)	0.75 (0.40, 1.39)
Social support at work	0.74 (0.48, 1.13)	0.84 (0.54, 1.30)	0.86 (0.56, 1.34)	0.88 (0.57, 1.37)
4) Occupational class:				
Managers and professionals	1.00			1.00
Semi-professionals	1.63 (0.93, 2.85)			1.37 (0.77, 2.46)
Routine non-manual workers	3.35 (1.86, 6.02)			2.78 (1.43, 5.42)
Manual workers	3.10 (1.93, 4.99)			1.75 (0.89, 3.46)

Hazard ratios (HR) and their 95% confidence intervals (95% CI). Women (n = 5122) and men (n = 1403).

First, among men, in the unadjusted base models the same working conditions as among women, except for social support, i.e. shift work, hazardous exposures, physical work load and job control were associated with all-cause disability retirement (Table 2). Also among men, the hazard ratio was highest for physical workload (HR 2.80, 95% CI 1.72-4.54). Second, in the groupwise models, after adjusting for specific working conditions within each broad subdomain of working conditions, the associations attenuated but remained. Third, in the models, mutually adjusting all working conditions for each other, physical workload remained clearly associated with disability retirement. The point estimates for hazardous exposures and job control attenuated substantially.

In addition, occupational class had a strong association with all-cause disability retirement among both women and men throughout the analyses. Adjusting for occupational class on top of all working conditions, the association of physical workload remained among women and men, as did job control among women. Among men an association of computer work emerged.

### Cause-specific disability retirement

In the cause-specific analyses for musculoskeletal diseases and mental disorders, women and men were pooled together, adjusting for gender. Gender interactions were controlled but none were found.

Adjusting first for gender only in the base model shift work, hazardous exposures, heavy physical workload and low job control were associated with disability retirement due to musculoskeletal diseases. The highest hazard ratio for retirement due to musculoskeletal diseases in Table 3 was for physical workload (HR 3.60, 95% CI 2.69-4.81). There were reverse associations for those working overtime, doing computer work and those with social support at their workplace. Second, after groupwise adjustments, the associations remained largely similar, but those for hazardous exposures and social support attenuated. Third, in the models, after mutually adjusting all working conditions for each other, physical workload and job control remained associated with disability retirement due to musculoskeletal diseases. Occupational class had a very strong own association. Adjusting for occupational class on top of all working conditions, the associations of physical workload and job control attenuated but remained.

**Table 3:** Associations of working conditions with disability retirement due to musculoskeletal diseases and mental disorders, adjusted for gender, groups of working conditions (=Groupwise), all working conditions and all variables in analysis

**Musculoskeletal diseases**	**Gender adjusted**	**Groupwise adjusted**	**All working conditions adjusted**	**Fully adjusted**
	**HR (95% CI)**	**HR (95% CI)**	**HR (95% CI)**	**HR (95% CI)**
1) Work arrangements:				
Shift work	1.85 (1.36, 2.52)	1.80 (1.32, 2.45)	1.13 (0.81, 1.56)	1.04 (0.75, 1.44)
Temporary work contract	0.58 (0.34, 1.01)	0.57 (0.33, 0.97)	0.60 (0.35, 1.03)	0.70 (0.40, 1.20)
Working overtime	0.52 (0.33, 0.83)	0.55 (0.35, 0.88)	0.73 (0.46, 1.18)	0.85 (0.53, 1.36)
2) Physical working conditions:				
Hazardous exposures	2.34 (1.75, 3.14)	1.43 (1.04, 1.98)	1.34 (0.97, 1.86)	1.16 (0.84, 1.61)
Physical workload	3.60 (2.69, 4.81)	3.00 (2.17, 4.14)	2.61 (1.88, 3.61)	2.10 (1.52, 2.89)
Computer work	0.62 (0.43, 0.90)	0.82 (0.56, 1.21)	0.83 (0.56, 1.23)	1.10 (0.73, 1.65)
3) Psychosocial working conditions:				
Low job control	2.61 (1.99, 3.42)	2.44 (1.85, 3.22)	1.96 (1.47, 2.61)	1.44 (1.07, 1.93)
High job demands	0.78 (0.55, 1.10)	0.84 (0.60, 1.19)	0.83 (0.58, 1.19)	0.94 (0.65, 1.34)
Social support at work	0.60 (0.43, 0.82)	0.69 (0.50, 0.96)	0.74 (0.53, 1.02)	0.80 (0.58, 1.11)
4) Occupational class:				
Managers and professionals	1.00			1.00
Semi-professionals	4.36 (2.32, 8.19)			3.43 (1.80, 6.53)
Routine non-manual workers	5.73 (3.24, 10.12)			3.64 (1.99, 6.66)
Manual workers	14.79 (8.39, 26.07)			7.83 (4.17, 14.68)
**Mental disorders**	**Gender adjusted**	**Groupwise adjusted**	**All working conditions adjusted**	**Fully adjusted**
	**HR (95% CI)**	**HR (95% CI)**	**HR (95% CI)**	**HR (95% CI)**
1) Work arrangements:				
Shift work	1.31 (0.87, 1.99)	1.32 (0.87, 2.00)	1.08 (0.70, 1.68)	1.03 (0.66, 1.60)
Temporary work contract	0.96 (0.56, 1.64)	0.96 (0.56, 1.63)	1.05 (0.61, 1.79)	1.06 (0.61, 1.81)
Working overtime	1.03 (0.66, 1.60)	1.05 (0.67, 1.63)	1.04 (0.66, 1.65)	1.12 (0.71, 1.78)
2) Physical working conditions:				
Hazardous exposures	1.62 (1.11, 2.36)	1.48 (0.98, 2.25)	1.35 (0.88, 2.06)	1.39 (0.91, 2.14)
Physical workload	1.57 (1.07, 2.31)	1.48 (0.95, 2.28)	1.36 (0.88, 2.10)	1.26 (0.80, 1.97)
Computer work	1.53 (1.06, 2.21)	1.71 (1.17, 2.50)	1.59 (1.08, 2.35)	1.60 (1.08, 2.38)
3) Psychosocial working conditions:				
Low job control	1.92 (1.34, 2.75)	1.93 (1.34, 2.78)	1.74 (1.19, 2.54)	1.67 (1.12, 2.49)
High job demands	1.52 (1.05, 2.19)	1.61 (1.11, 2.32)	1.43 (0.97, 2.09)	1.48 (1.00, 2.18)
Social support at work	0.73 (0.51, 1.07)	0.81 (0.55, 1.18)	0.81 (0.56, 1.19)	0.82 (0.56, 1.19)
4) Occupational class:				
Managers and professionals	1.00			1.00
Semi-professionals	1.69 (1.03, 2.76)			1.64 (0.98, 2.74)
Routine non-manual workers	1.85 (1.19, 2.87)			1.64 (0.99, 2.73)
Manual workers	1.38 (0.79, 2.42)			1.07 (0.55, 2.09)

Hazard ratios (HR) and their 95% confidence intervals (95% CI) (n = 6525).

For disability retirement due to mental disorders, adjusting first for gender only in the base model, hazardous exposures, physical workload and computer work, as well as low job control and high job demands, showed associations. For retirement due to mental disorders, the highest hazard ratio was for low job control (HR 1.92, 95% CI 1.34-2.75). Second, after groupwise adjustments, computer work as well as job control and job demands remained associated with disability retirement due to mental disorders. Third, mutually adjusting for all working conditions for each other, led to only somewhat attenuated associations. Adjusting for occupational class on top of all working conditions only slightly affected the associations found.

Further analyses were made for disability retirement due to all causes other than musculoskeletal and mental, pooling women and men (no data shown). Of the working conditions, only physical workload and additionally occupational class were associated with disability retirement throughout these analyses.

## Discussion

This study sought to examine the associations of work arrangements, physical working conditions and psychosocial working conditions with subsequent disability retirement due to all causes as well as musculoskeletal and mental causes. Survey data on working conditions were linked with register data on retirement over a six to eight year follow up.

The main findings were:

1) Overall, the studied physical and psychosocial working conditions had some associations with disability retirement. Several specific working conditions showed own associations, but after considering all working conditions simultaneously, heavy physical workload and low job control remained the primary risk factors for disability retirement.

2) Work arrangements were inconsistently associated with disability retirement. Own associations were partly contrasting and after other working conditions were considered, work arrangements were no longer associated with disability retirement.

3) Disability retirement due to all causes, as well as musculoskeletal causes shared similar risk factors. In accordance with our expectations the strongest risk factor for musculoskeletal causes was heavy physical workload while for mental causes the main risk factor was low job control.

4) Gender differences in the risk factors for all-cause disability retirement could not be confirmed.

5) Additionally included occupational class was associated with disability retirement in general and musculoskeletal causes in particular. Nevertheless, the effects of class on the work-related risk factors for disability retirement were modest.

### Interpretation

Work dis/ability is ultimately based on both health-related resources and requirements posed by the working environment. Our focus was on the working environment with the aim of detecting which, among the specific working conditions, are the primary risk factors contributing to disability retirement independent of other working conditions.

Many of the specific working conditions showed associations with disability retirement, but after adjustments the associations were few. A major finding was that heavy physical workload was a strong risk factor for disability retirement independent of all other working conditions throughout the analyses, except for retirement due to mental disorders. A number of previous studies have also shown the importance of physical exposures to work disability [12,14,18,26]. However, these studies have failed to simultaneously consider other subdomains of working conditions. Our study thus contributes to the previous evidence by highlighting that heavy physical workload over and above other working conditions continues to be an important driving force for disability retirement among the studied employees. To reach this conclusion, a comprehensive work environment framework was needed.

Among the psychosocial working conditions low job control was the most consistent risk factor for disability retirement and that concerned also mental disorders. Additionally, only high job demands showed a modest independent association with disability retirement due to mental disorders.

In previous studies, psychosocial factors such as mental strain, job dissatisfaction and low social support [12], lack of decision authority [8] and autonomy [14], job strain [17] and burnout [16] have been associated with disability retirement, although not fully consistently. However, focusing exclusively on psychosocial working conditions, these previous studies have overlooked the potential importance of other working conditions.

Work arrangements have received little previous study as risk factors for disability retirement, and the existing evidence is heterogeneous. Organisational changes [10] as well as long working hours [12] have shown associations with disability retirement. However, employees in regular day-time work have been shown to be at a higher risk of retirement than those doing shift work [12]. In our study none of the work arrangements remained associated with disability retirement throughout the analyses.

The socioeconomic indicator used by us was occupational class, which is a known risk factor for disability retirement [27-29]. This was reconfirmed and a very strong association was found for retirement due to musculoskeletal causes. Considering occupational class on top of all working conditions had but minor effects on the associations, with some attenuating and others strengthening, but none of the previous associations disappearing completely. Our analyses also reconfirmed that occupational class is a clear risk factor for disability retirement independent of all studied working conditions. It has previously been reported that there are occupational class differences in musculoskeletal causes and to a lesser degree in mental causes of disability retirement [30,31], but in our study, consistent differences for mental disorders could not be confirmed. Sensitivity analyses were made to check residual confounding by household income. Income was a less powerful socioeconomic determinant but it shared some own associations with disability retirement besides occupational class. However, the effects of income on the main associations under study, i.e. between working conditions and disability retirement, were minor, with income strengthening rather than weakening the identified associations.

Further sensitivity analyses were made to control for the effect of baseline health on the studied associations. This was accomplished by adjusting the analyses for self-rated health, which is a strong determinant of disability retirement [32]. After adjusting for baseline self-rated health, the associations of working conditions with subsequent disability retirement weakened somewhat, and lost statistical significance among men and for mental disorders, partly due to the small number of retirement events. However, it is doubtful whether baseline health should be understood only as a confounder in our analyses. The associations between working conditions and disability retirement are highly complex, and baseline health is also likely to represent the pathway to further ill-health, diagnosis and work disability ultimately leading to disability retirement. Thus the inclusion of baseline health in the analyses may run the risk of over-adjustment.

For the main medical causes of disability retirement we were able to examine two major diagnostic groups - musculoskeletal diseases and mental disorders. The work-related risk factors for disability retirement due to all causes, as well as musculoskeletal causes were very similar. This is understandable as musculoskeletal disorders are the largest diagnostic cause for disability retirement. Mental disorders also account for a substantial part of all disability retirement events, but the risk factors for mental disorders, independent of other working conditions, consisted of computer work and low job control, the latter being one of the two dimensions of Karasek’s [23] job strain. Among the working conditions, physical exposures were most pronounced for somatic causes, whereas the psychosocial exposures were most pronounced for mental causes of disability retirement as we expected. For causes other than musculoskeletal or mental, only physical workload was associated with disability retirement.

Gender differences in the risk factors could be examined for all-cause disability retirement only. The risk factors for both genders were similar throughout the analyses. However, for various medical causes of disability retirement, the gender differences in the risk factor profiles are not necessarily similar. Further research on gender differences in disability retirement is warranted.

Comparing associations of work-related risk factors for disability retirement from the present study with previous ones is complicated, as typically the focus has been on one particular factor or one particular subdomain of working conditions at a time. A number of studies have examined psychosocial factors, but simultaneously overlooked physical working conditions. Thus the relative importance of each working condition cannot be judged. Our study confirms that examining work-related risk factors for disability retirement benefits from a multi-domain framework, allowing for simultaneous analyses of various types of specific working conditions ranging from work arrangements to psychosocial and physical working conditions.

### Methodological considerations

The strengths of our study include a longitudinal design and retirement data derived from complete national registers with diagnoses for disability retirement. A further strength is the variety of working conditions from different subdomains. However, self-reported information on working conditions may be susceptible to reporting bias.

Participation in the baseline survey was acceptable and we were able to conduct extensive non-response analyses [20,21]. Judging from these analyses, it is unlikely that the non-participation would substantially bias the associations between working conditions and disability retirement. Nevertheless, we acknowledge that the non-response was substantial and remains a potential source of bias.

We had cause-specific data on disability retirement, but the number of events was limited and only allowed the two largest diagnostic groups i.e. musculoskeletal diseases and mental disorders, to be separately analysed. Gender stratification in the cause-specific analyses was not possible.

Finally we studied an occupational cohort from the public sector, and our findings may differ from the private sector. In a Danish study the risk of disability retirement was higher among women from the public sector [33]. Thus generalisations about the total workforce in Finland or other countries are not warranted.

## Conclusions

Our study confirms that working conditions are potential risk factors for disability retirement. Among the various work-related factors, physically heavy work is likely to feature prominently. Low job control is an additional risk factor. Thus the physical work environment and the opportunity to control one’s own job, provide areas for interventions and policies to prevent early retirement due to disability. Successful prevention would contribute to longer working careers, lower costs of work disability, as well as healthy ageing and old-age retirement in good health.

## Competing interests

The authors declare that they have no competing interests.

## Authors’ contributions

EL drafted the manuscript. The study and its analyses were designed together with the co-authors, who commented on the drafts and contributed to the writing of the manuscript. OP carried out the statistical analyses. All authors read and approved the final manuscript.

## Pre-publication history

The pre-publication history for this paper can be accessed here:

http://www.biomedcentral.com/1471-2458/12/309/prepub
